# *Listeria* Endocarditis: A Diagnostic Challenge

**DOI:** 10.1177/2324709617698995

**Published:** 2017-04-10

**Authors:** Wilhelmina J. A. R. M. Valckx, Suzanne P. M. Lutgens, Hortence E. Haerkens-Arends, Peter C. Barneveld, Jaap J. Beutler, Ellen K. Hoogeveen

**Affiliations:** 1 Jeroen Bosch Hospital, Den Bosch, the Netherlands

**Keywords:** endocarditis, *Listeria monocytogenes*, ^18^F-FDG PET-CT scan

## Abstract

A 74-year-old hemodialysis patient with a history of an atrial septum defect closure, coronary bypass surgery, and a St. Jude aortic prosthetic valve was diagnosed with pneumonia and volume overload. Blood cultures were positive for *Listeria monocytogenes*, and amoxicillin was given for 2 weeks. Immediately after discontinuation of amoxicillin, fever relapsed. Transthoracic and transesophageal echocardiography showed no sign of endocarditis. Given the fever relapse and 3 positive minor Duke criteria, an ^18^F-FDG PET-CT scan (^18^F-fluorodeoxyglucose-positron emission tomography-computed tomography) scan was performed. This scan showed activity at the aortic root, proximal ascending aorta, and inferior wall of the heart, making *Listeria monocytogenes* endocarditis a likely explanation. Amoxicillin was given for 6 weeks with good clinical result. Diagnosing a life-threatening *Listeria monocytogenes* endocarditis can be challenging and an ^18^F-FDG PET-CT scan can be helpful.

## Introduction

In the general population, the bacterium *Listeria monocytogenes* causes infections with an annual incidence of 0.32 cases per 100 000 inhabitants in Europe. Of all the zoonotic diseases under European Union surveillance, listeriosis represents the most severe human disease in terms of hospitalization and case fatality rate; in 2011, 94% of identified cases were hospitalized, with a case fatality rate of 13%.^1^ Especially in the elderly, neonates, and cell-mediated immunosuppressed patients, *Listeria monocytogenes* can cause life-threatening infections.^2^ In this case report, we present a patient with an extraordinary diagnostic course of *Listeria* endocarditis, an infection affecting about 8% of the *Listeria*-infected adults.^3^

## Case Report

**Figure 1.**
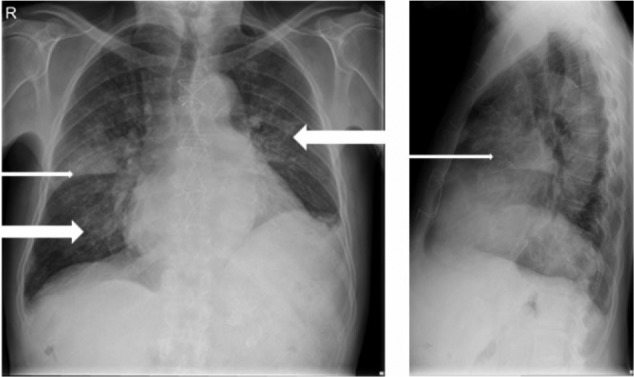
Chest radiography Anteroposterior (left) and sagittal (right). The thin arrows point at the consolidation of the right upper lobe and the bold arrows point at the redistribution.

A 74-year-old hemodialysis patient with a history of atrial septum defect closure, coronary bypass surgery, and a St. Jude aortic prosthetic valve for 13 years was admitted through the dialysis unit because of coughing without sputum and dyspnea. The temperature was not measured at home, but the patient might have had chills. During physical examination the blood pressure was 153/74 mm Hg, pulse 74 beats/minute, temperature 37°C, saturation 100% with 2 L/min oxygen supply, and a respiratory rate of 16/minute. During auscultation the prosthetic heart valve was clearly audible without any other murmurs. Auscultation of the lungs revealed on the right midthoracic side decreased breath sounds and crackles on the lower posterior lung fields. Abdominal examination was normal. Pitting edema was present in both lower legs. Blood samples that were taken before hemodialysis that day showed the following: hemoglobin 5.4 mmol/L, mean corpuscular volume 100 fL, white blood cell count 11.9 × 10^9^/L, thrombocytes 9 × 10^9^/L, urea 59.9 mmol/L, creatinine 1100 µmol/L, potassium 6.6 mmol/L, bicarbonate 17 mmol/L; C-reactive protein (CRP) 48 mg/L, lactate dehydrogenase 465 U/L, and NT-pro BNP 175 000 ng/L. Chest radiography revealed pulmonary congestion and a consolidation of the right upper lobe (Figure 1).

Based on these results, the patient was diagnosed with fever and dyspnea owing to pneumonia and volume overload. Two blood culture sets were taken simultaneously before start of hemodialysis, and the patient was treated empirically with the antibiotics ciprofloxacin and penicillin intravenously and extra ultrafiltration during hemodialysis. The maximum body temperature was 38.2°C. Because both blood cultures yielded positive for *Listeria monocytogenes*, an additional blood culture set was taken 2 days later and a transthoracic echocardiography performed. Echocardiography showed no prosthetic valve dysfunction or valvular vegetations and a reasonable systolic left ventricle ejection fraction, but moderate right ventricular function. Because of these findings, the clinical presentation and the presence of only 3 minor Duke criteria (one blood culture with an atypical microorganism, a predisposing heart condition, and fever), infective endocarditis was considered unlikely. The patient was treated for *Listeria* pneumonia with the antibiotic amoxicillin for 2 weeks and had initially a good clinical response, a decrease of CRP (maximum rise was 177 mmol/L), and normalization of the thrombocytes.

**Figure 2.**
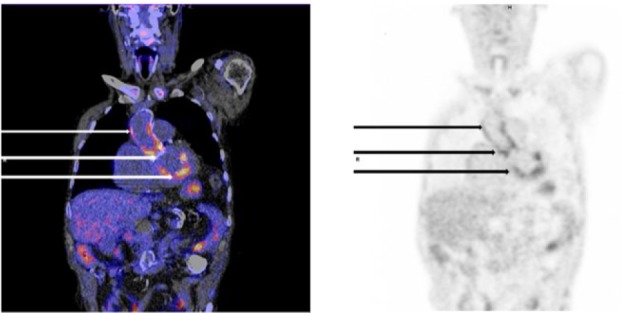
^18^F-FDG PET scan and CT scan Coronal view of the ^18^F-FDG PET in fusion with low dose CT (left) and only ^18^FDG PET (right) showing activity at the proximal ascending aorta (upper arrows), aortic root (middle arrows) and inferior wall of the heart (lower arrows).

However, immediately after discontinuation of treatment with amoxicillin, fever relapsed with a rise of CRP. Therefore, we reconsidered the diagnosis *Listeria* endocarditis. This time a transesophageal echocardiography was performed, but it showed no valvular insufficiency or valve vegetations and a good left and right ventricular function. Therefore, an ^18^F-FDG PET-CT scan (^18^F-fluorodeoxyglucose-positron emission tomography-computed tomography) scan was performed. This scan showed activity at the aortic root, proximal ascending aorta, and inferior wall of the heart (Figure 2). Given the relapse of fever, increasing CRP level after discontinuation of amoxicillin, the 3 positive minor Duke criteria, and the positive ^18^F-FDG PET-CT scan, *Listeria monocytogenes* endocarditis was the presumptive diagnosis. We treated the patient with amoxicillin for 6 weeks with a good clinical result. After these 6 weeks of treatment with antibiotics, the fever relapsed for a second time and the clinical condition of the patient rapidly deteriorated. There were no neurological findings. According to his wishes, we performed no further diagnostic tests like an ^18^F-FDG PET-CT scan or cardiac echocardiography to compare with the former ones or other imaging techniques to look for embolic events. His poor clinical condition prohibited an aortic valve replacement, which also was not desired by the patient. We advised lifelong treatment with oral amoxicillin, but this was refused by the patient. The patient was discharged home following his request, where he died shortly thereafter. Unfortunately, postmortem autopsy to confirm the diagnosis of *Listeria* endocarditis was not performed.

## Discussion

*Listeria monocytogenes* is a facultative anaerobic, nonspore-forming gram-positive rod that can be isolated from soil, dust, animal feed, water, sewage, and the tissues or fluid of almost any type of animal. Frequently, it can be found in raw and unprocessed foods such as meats and dairy products. Hospital-associated infections are reported sporadically.^3-6^

*Listeria monocytogenes* is a transient colonizer of the human gastrointestinal tract that causes no symptoms. It can be isolated in 1% to 10% of the general population’s stool. If invasive, this bacterium can penetrate host cells through (induced) phagocytosis. Once intracellular, *Listeria monocytogenes* uses “listeriolysin O” to avoid intracellular killing. Subsequently, it invades adjacent cells through plasma membrane protrusions. This way, this bacterium avoids the extracellular human T-cell immune system and antibiotics. Immune incompetent hosts, like elderly, neonates, pregnant women, and patients who use immunosuppressive medication, cannot activate macrophages to destroy this bacterium.^2,4^

The incidence of infective endocarditis is about 3 to 10 episodes/100 000 person-years and increases with age.^7^ Prosthetic valve endocarditis occurs in 1% to 6% of all patients with valve protheses.^8^ The prognosis is influenced by comorbidity, cardiac and noncardiac complications, echocardiographic findings, and the infecting organism. The in-hospital mortality risk is about 15% to 30% and 20% to 40% for prosthetic valve endocarditis. The survival rates after completion of treatment are estimated to be 80% to 90% at 1 year, 70% to 80% at 2 years, and 60% to 70% at 5 years.^8^ The incidence and mortality rates of *Listeria* endocarditis are not known. Endocarditis is observed in about 8% of adults infected with *Listeria monocytogenes* and occurs on both native and prosthetic valves.^3,9^ Antolín et al published in 2008 that since the first description in 1955, they only found 68 published case reports of *Listeria* endocarditis. Of these 68 patients, 24 died (35%), most of them before 1985. Since then case fatality has fallen to 12%, presumably because of an increase in indication for surgery and the improved results, according to Antolín et al.^9^

The modified Duke criteria are useful for diagnosing infective endocarditis (sensitivity and specificity 80%, when evaluated at the end of follow-up in epidemiological studies), but they do not replace clinical judgment. They are based on clinical and biological findings, transthoracic and/or transesophageal echocardiography, blood cultures, and serology.^7,8^ Our case report illustrates, however, that the modified Duke criteria may have a lower diagnostic accuracy for early diagnosis in clinical practice. For instance, in patients with prosthetic valve, pacemaker, or defibrillator lead infective endocarditis, echocardiography is normal or inconclusive in up to 30% of cases.^8^ The Task Force for the Management of Infective Endocarditis of the European Society of Cardiology state in their newest guideline that echocardiography, positive blood cultures, and clinical features remain the cornerstone of diagnosis. If, however, the diagnosis of endocarditis remains “possible” or even “rejected,” but there is a persisting high level of clinical suspicion, echocardiography and blood culture should be repeated and other imaging techniques should be performed, for instance, cardiac CT, ^18^F-FDG PET-CT scan, or radiolabelled leucocyte SPECT/CT. In case of embolic events an cerebral magnetic resonance imaging, whole-body CT, and/or PET/CT is advised.^8^

The Task Force states that an ^18^F-FDG PET-CT scan has been shown to be particularly useful for the diagnosis of prosthetic valve endocarditis.^8^ According to a review of Bruun et al in 2014, the sensitivity of the modified Duke criteria at admission increases from 70% to 97% when adding an abnormal ^18^F-FDG PET-CT scan at the location of a prosthetic valve as a new major criterion.^10^

Because of the severity of complications and high mortality risk, treatment of a *Listeria* infection should be started as soon as possible. The recommended choice of treatment is a β-lactam antibiotic, such as amoxicillin. Because the penicillins are bacteriostatic, simultaneous use of, for example, an aminoglycoside (usually gentamicin), can be necessary.^2^ According to the Dutch national guidelines, treatment of *Listeria* bacteremia should extend for 3 weeks.^11^

In conclusion, *Listeria monocytogenes* endocarditis can be life-threatening. Therefore, early treatment with a β-lactam antibiotic is important. However, establishing the diagnosis of *Listeria* endocarditis can be challenging. This case report shows that the ^18^F-FDG PET-CT scan can be useful to support the diagnosis of endocarditis, in accordance with the newest guideline of the Task Force for the Management of Infective Endocarditis of the European Society of Cardiology.
